# Indirubin, a bisindole alkaloid from *Isatis indigotica*, reduces H1N1 susceptibility in stressed mice by regulating MAVS signaling

**DOI:** 10.18632/oncotarget.22350

**Published:** 2017-11-09

**Authors:** Chong Jie, Zhuo Luo, Huan Chen, Min Wang, Chang Yan, Zhong-Fu Mao, Gao-Keng Xiao, Hiroshi Kurihara, Yi-Fang Li, Rong-Rong He

**Affiliations:** ^1^ Anti-Stress and Health Research Center, College of Pharmacy, Jinan University, Guangzhou 510632, China; ^2^ Institute of Traditional Chinese Medicine & Natural Products, Jinan University, Guangzhou 510632, China; ^3^ Department of Pharmacy, Hainan General Hospital, Hainan 570311, China

**Keywords:** indirubin, influenza virus, virus susceptibility, MAVS, STING

## Abstract

*Isatis indigotica* has a long history in treating virus infection and related symptoms in China. Nevertheless, its antivirus evidence in animal studies is not satisfactory, which might be due to the lack of appropriate animal model. Previously, we had utilized restraint stress to establish mouse H1N1 susceptibility model which was helpful in evaluating the anti-virus effect of medicines targeting host factors, such as type I interferon production. In this study, this model was employed to investigate the effect and mechanism of indirubin, a natural bisindole alkaloid from *Isatis indigotica*, on influenza A virus susceptibility. In the *in vitro* study, the stress hormone corticosterone was used to simulate restraint stress. Our results demonstrated that indirubin decreased the susceptibility to influenza virus with lowered mortality and alleviated lung damage in restraint-stressed mice model. Moreover, indirubin promoted the expression of interferon-β and interferon inducible transmembrane 3. In addition, indirubin maintained the morphology and function of mitochondria following influenza A virus infection. Further study revealed that indirubin promoted interferon-β production through promoting mitochondrial antiviral signaling pathway. Our study indicated that indirubin could be a candidate for the therapy of influenza.

## INTRODUCTION

Influenza, as a highly contagious disease, poses a health threat to humans. According to an online report by Centers for Disease Control and Prevention, 310 thousand people were hospitalized because of influenza-related illness during the 2015-2016 influenza season [1]. At present, vaccine and antiviral drugs are the major countermeasures against influenza virus infection. However, vaccination needs to be conducted annually as the body's immune response from vaccine declines over time and influenza viruses constantly change [2]. Besides, the influenza vaccine is not suitable for people who are severely allergic to chicken eggs [3]. Moreover, current antiviral drugs including M2 and neuraminidase (NA) protein inhibitors would be limited by the appearance of resistant viruses [4]. In fact, apart from viral factors, host factors are also important during the process of influenza virus infection [5]. Pattern recognition receptors (PRRs) detect pathogen-associated molecular patterns (PAMPs), including influenza virus, which results in the production of type I interferons (IFNs) [6]. Type I IFNs exhibit a non-redundant effect. Type I IFNs inhibit virus replication, boost adaptive immunity and limit acute lung injury [7, 8]. Therefore, novel anti-influenza virus drugs targeting host factors is worth discovering. Our group previously had utilized restraint stress to disrupt type I IFN secretion and increase the susceptibility to H1N1 influenza virus. Based on this model, we have successfully evaluated the anti-influenza efficacy of several traditional Chinese medicines or natural products, such as apple polyphenols, *Sarcandra glabra* extract, and ReDuNing injection [9–11].

*Isatis indigotica*, a traditional medicine, is widely applied in clinic in China to treat viral diseases such as hepatitis, influenza and encephalitis [12, 13]. Recent studies also showed that it had antiviral activities against Japanese encephalitis virus, rabies, influenza A and some other viruses [12, 14–18]. Indirubin, a bisindole alkaloid, is the main active ingredient of *Isatis indigotica*. Mak and his colleagues proved the antiviral activity of indirubin against influenza by inhibiting the production of the chemokine regulated on activation, normal T cell expressed and secreted in human bronchial epithelial cells infected with influenza virus [17]. Chang's laboratory showed indirubin had an antiviral effect on Japanese encephalitis virus through blocking virus attachment [14]. However, these are *in vitro* studies, further *in vivo* studies are required. Indirubin also shows the anti-inflammatory effect as it has been reported to inhibit the inflammatory reactions of delayed-type hypersensitivity [19]. These previous studies suggest indirubin is a potential agent against influenza virus, but its underlying mechanism retains unclear. For such purpose, restraint-stressed mouse model along with stress hormone corticosterone (CORT)-loaded A549 cell model is employed to investigate the anti-influenza effect of indirubin and explore its mechanism.

## RESULTS

### Indirubin attenuates the morbidity and mortality caused by influenza infection in stressed mice

**Figure 1:**
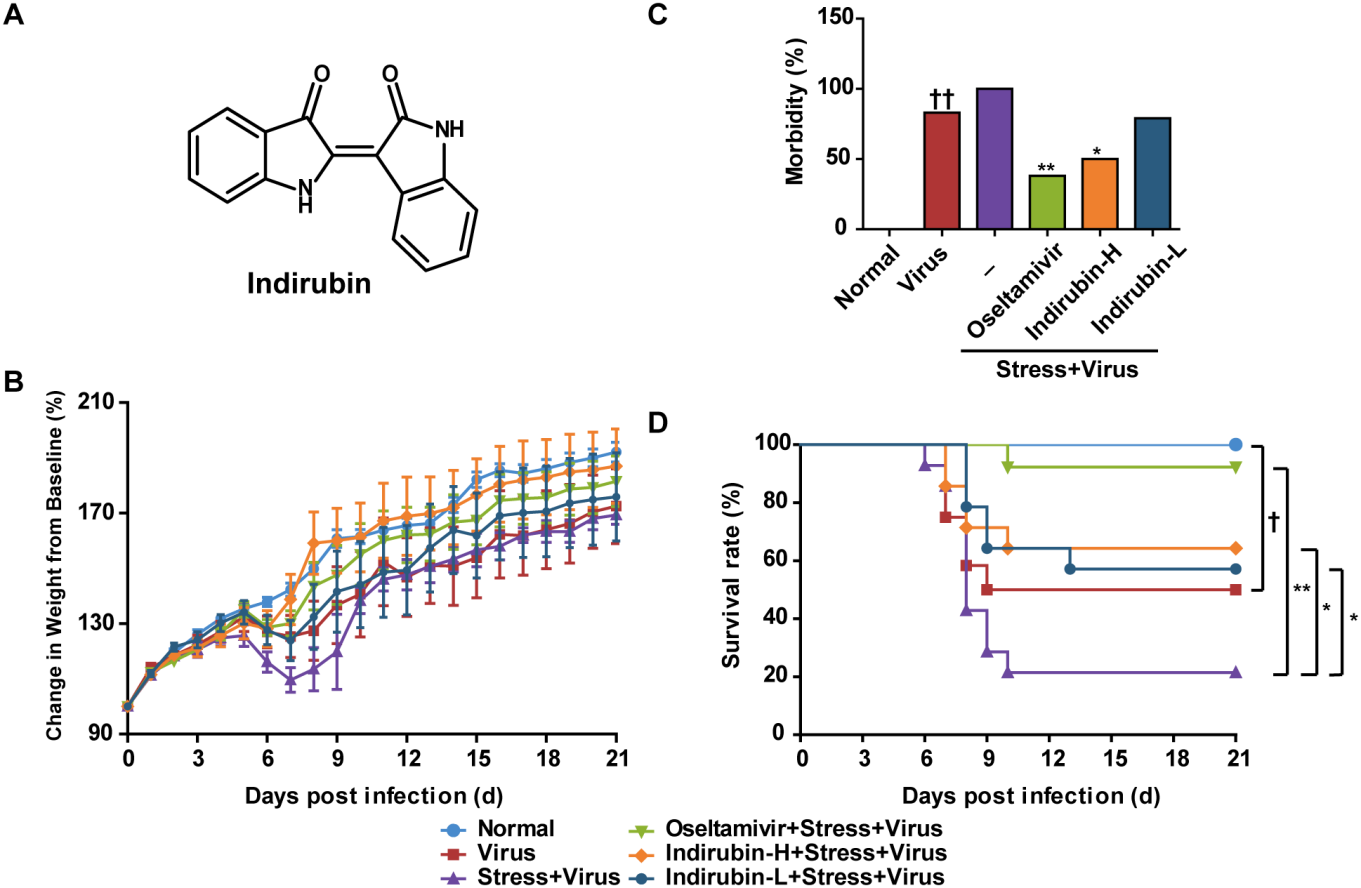
Indirubin attenuates the morbidity and mortality caused by influenza infection in stressed mice Chemical structure of indirubin **(A)**. Effects of indirubin on body weight changes **(B)**, morbidity **(C)** and survival rate **(D)** of mice after influenza infection. “-” indicates no treatment. Indirubin-H and Indirubin-L respectively represent the higher dose of indirubin (5 mg·kg^−1^·d^−1^) and the lower dose of indirubin (2.5 mg·kg^−1^·d^−1^). The difference was considered statistically significant at ^†^*P* < 0.05 vs. Virus group; ^*^P < 0.05 and ^**^P < 0.01 vs. “Stress+Virus” group. Data were obtained from 10 - 14 animals in each group.

After H1N1 infection by intranasal inoculation, body weight changes and survivals of mice in each group were monitored daily for 21 days. As illustrated in Figure 1B, the average body weight of mice in Virus group began to decrease on day 6 and dropped to a minimum on day 7, and gained weight on day 8. However, the average body weight of mice in the “Stress+Virus” group did not recover until day 10. Compared to the “Stress+Virus” group, oseltamivir, indirubin-H and indirubin-L treatment could significantly maintain the body weight of restraint-stressed mice infected with influenza virus. The morbidity of the mouse was estimated when its weight was decreased over 1 g·d^−1^. As shown in Figure 1C, compared to the Virus group, “Stress+Virus” group was observed a higher morbidity (83% vs. 100%). Compared with the “Stress+Virus” group, oseltamivir, indirubin-H and indirubin-L reduced the morbidity to 38% (*P* < 0.01), 50% (*P* < 0.05) and 79%, respectively. The data above indicated a role of indirubin on alleviating the morbidity of stressed mice infected with influenza virus, which was also supported by the improvement of behavioral changes, such as the altered respiration and ruffed fur. Then, we calculated the survivals and estimated the mortality of mice in each group. As shown in Figure 1D, a significantly lowered survival rate was observed in “Stress+Virus” group as compared with Virus group (*P* < 0.05). In comparison, oseltamivir, indirubin-H and indirubin-L treatment significantly improved the survival rate to 92% (*P* < 0.01), 64% and 57% (*P* < 0.05), respectively.

### Indirubin protects against pneumonia caused by influenza infection in stressed mice

**Figure 2:**
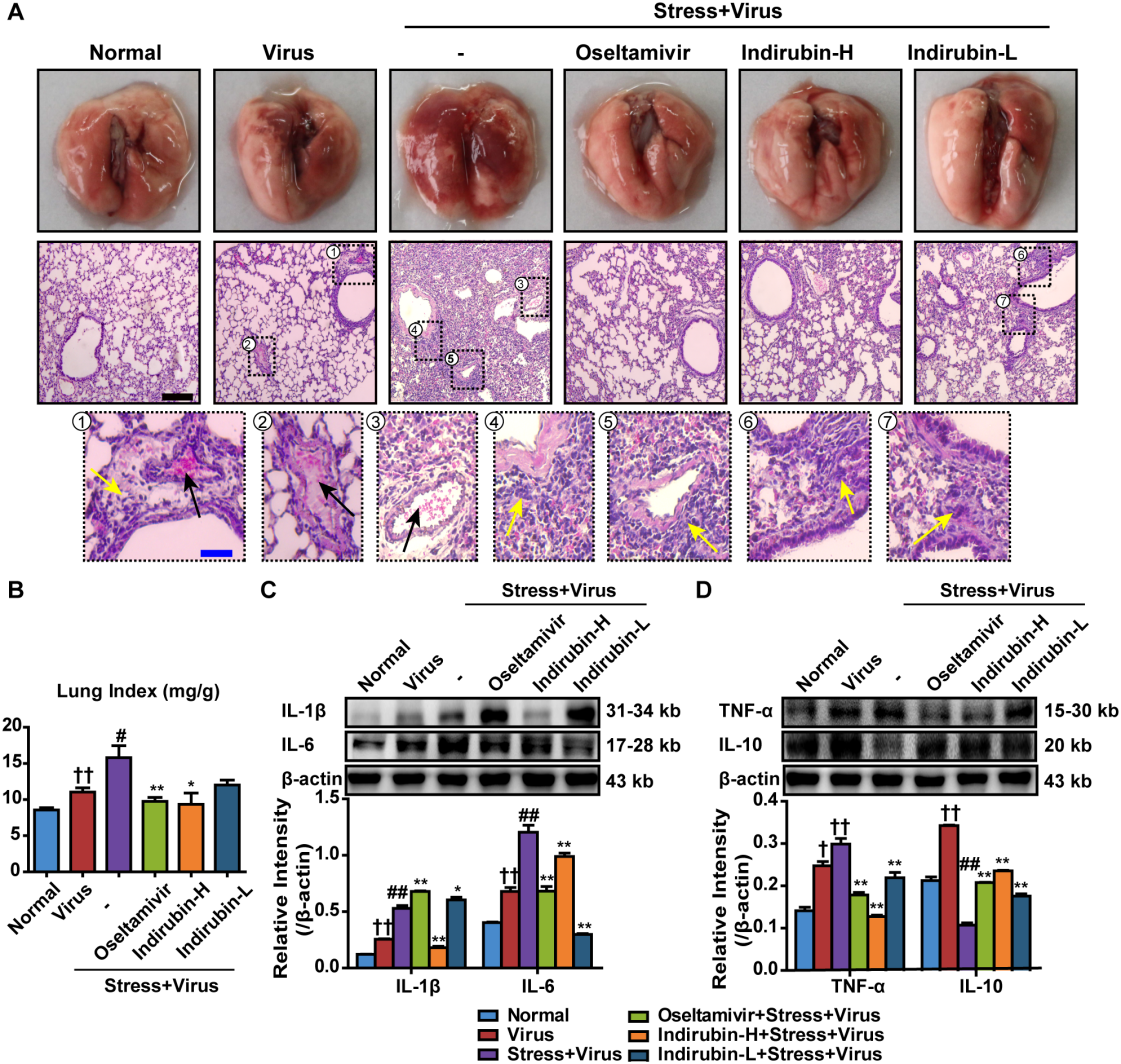
Indirubin protects against pneumonia caused by influenza infection in stressed mice **(A)** Histologic sections of lung tissues on the 5^th^ day after influenza virus challenge, stained by H&E to exam histopathologic changes (Black scale bar = 100 μm, Blue scale bar = 25 μm). Black arrows represent congesting vessels and yellow arrows stand for inflammatory cells infiltration. **(B)** Effects of indirubin administrations on the lung index in H1N1-infected restraint-stressed mice. The protein expressions of IL-1β, IL-6 **(C)**, TNF-α and IL-10 **(D)** in the lung tissues. “-” indicates no treatment. Indirubin-H and Indirubin-L respectively represent the higher dose of indirubin (5 mg·kg^−1^·d^−1^) and the lower dose of indirubin (2.5 mg·kg^−1^·d^−1^). The difference was considered statistically significant at ^†^*P* < 0.05, ^††^*P* < 0.01 vs. Normal group; ^#^*P* < 0.05, ^##^*P* < 0.01 vs. Virus group; ^*^P < 0.05, ^**^P < 0.01 vs. “Stress+Virus” group. Data are expressed as mean ± SEM from 3 independent determinations.

Severe pneumonia primarily accounts for the morbidity and mortality after influenza infection. The lung tissues were removed on the 5^th^ day after influenza virus challenge to detect the pathological changes. As shown in Figure 2A, edema and extravasation of red blood cell were seen in fresh lung tissues of mice in the “Stress+Virus” group. H&E staining showed that some alveoli were filled with exudates and congesting vessels were observed in the lung tissues of stressed mice following H1N1 infection. By contrast, oseltamivir, indirubin-H and indirubin-L significantly alleviated the lung pathogeneses with slight lesions. We then estimated the pulmonary edema by calculating the lung index in each group. As shown in Figure 2B, the lung index of the Normal group was 8.6 ± 0.75 mg·g^−1^. It increased to 11.0 ± 1.3 mg·g^−1^ (*P* < 0.01) in Virus group and further increased to 15.8 ± 4.4 mg·g^−1^ (*P* < 0.05) in “Stress+Virus” group. Nevertheless, oseltamivir, indirubin-H and indirubin-L notably decreased it to 9.7 ± 1.4 mg·g^−1^ (*P* < 0.01), 9.3 ± 4.1 mg·g^−1^ (*P* < 0.05) and 12.0 ± 1.8 mg·g^−1^, respectively.

Pro-inflammatory responses are essential for the early control of influenza viral replication. However, excessive inflammation results in tissue damaging cytotoxic in the lungs [20]. Western blotting was employed to detect the levels of pro-inflammatory cytokine. As shown in Figure 2C and Figure 2D, the expression levels of interleukin-1β (IL-1β), IL-6 and TNF-α of lungs in “Stress+Virus” group both significantly increased, when compared to Virus group. However, a modest attenuation of IL-1β occurred in the groups of indirubin-H and indirubin-L treatment. Meanwhile, indirubin-H and indirubin-L decreased IL-6 and TNF-α production in the lung tissues following H1N1 infection (*P* < 0.01). In contrast, indirubin treatment induced an elevated production of IL-10, a pleiotropic cytokine limiting inflammatory responses, in the lung tissues of stressed mice after virus infection (*P* < 0.01). These results showed that indirubin navigated a balance of pro-inflammatory responses and protected against pneumonia associated with influenza infection in restraint-stressed mice.

### Indirubin promotes IFN-β generation through MAVS antiviral signaling after influenza infection in stressed mice

**Figure 3:**
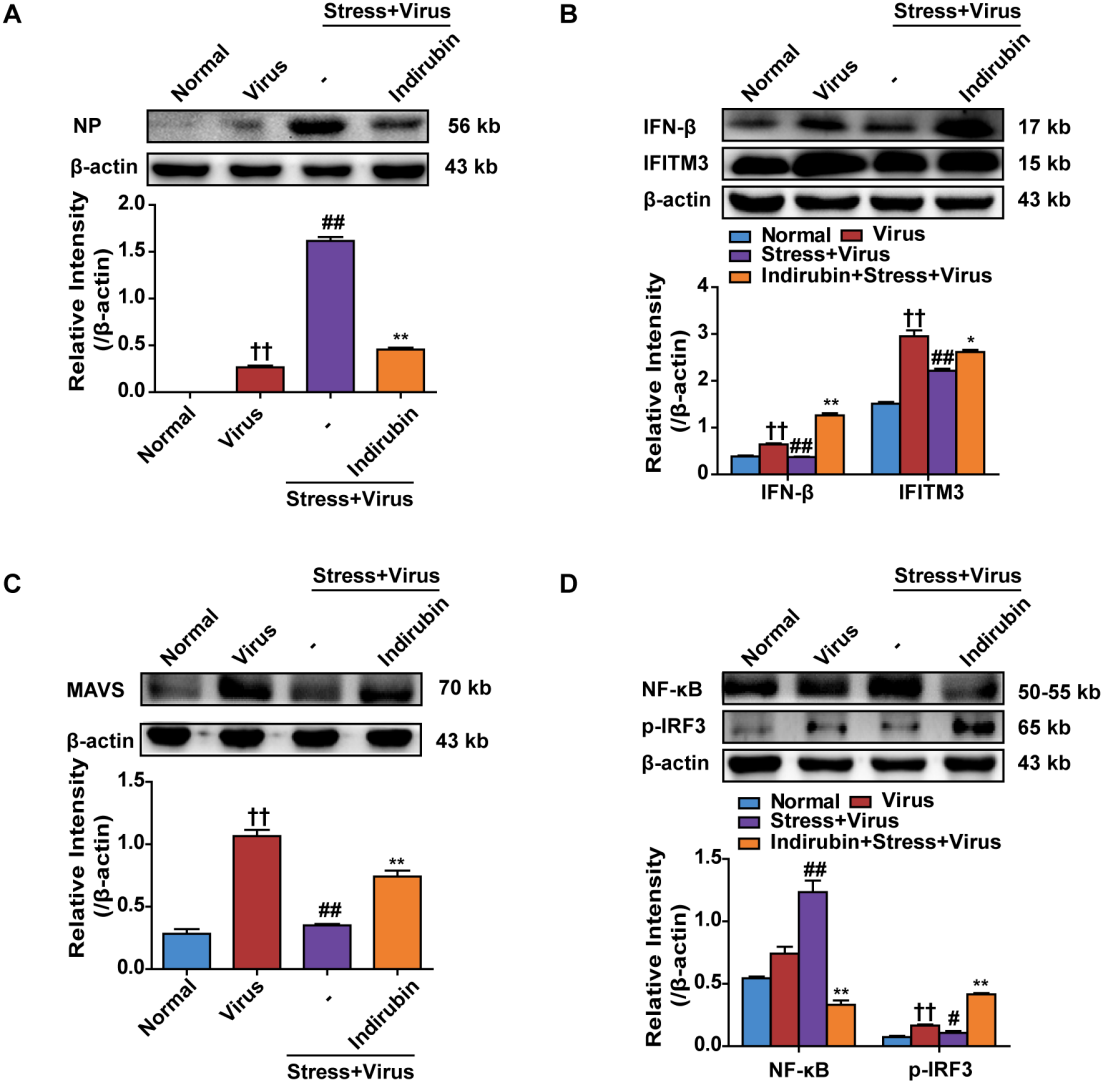
Indirubin promotes IFN-β generation through MAVS antiviral signaling after influenza infection in stressed mice The protein expressions of NP **(A)**, IFN-β, IFITM3 **(B)**, MAVS **(C)** and p-IRF3, NF-κB **(D)** in the lung tissues on the 5^th^ day post infection. “-” indicates no treatment. The difference was considered statistically significant at ^††^*P* < 0.01 vs. Normal group; ^#^*P* < 0.05, ^##^*P* < 0.01 vs. Virus group; ^*^P < 0.05, ^**^P < 0.01 vs. “Stress+Virus” group. Data are expressed as mean ± SEM from 3 independent determinations.

Nucleoprotein (NP) level indicates the replication state of influenza virus. As shown in Figure 3A, NP protein expression in lungs of “Stress+Virus” group increased remarkably (*P* < 0.01), comparing with Virus group. However, indirubin treatment obviously reduced NP protein level (*P* < 0.01). Influenza virus is recognized by host sensors, which results in type I IFNs secretion [6]. Type I IFNs, including IFN-β, activate innate antiviral responses to suppress virus replication. Besides, IFN-β could induce the expression of interferon inducible transmembrane 3 (IFITM3), which inhibits viral RNA from translocating into nuclear for replication and appreciation [21]. In the present study, both IFN-β and IFITM3 protein expressions were obviously decreased in the lung tissues in stressed mice following H1N1 infection, which was inhibited by indirubin treatment (Figure 3B). We next explored how indirubin induced IFN-β production and paid attention to MAVS signaling. The detection of virus RNA by RIG-I, the essential PRR within cells, will lead to the aggregation of MAVS. This signaling results in the activation of NF-κB and interferon regulatory factor 3 (IRF3), which respectively stimulated the production of pro-inflammatory cytokines and type I IFNs [22]. In our study, we found that the protein expressions of MAVS and phosphorylation of IRF3 declined, while the protein expression of NF-κB increased in the lungs of mice in “Stress+Virus” group (*P* < 0.01), comparing with Virus group. Indirubin treatment elevated MAVS expression and promoted the phosphorylation of IRF3 in the lung tissues (Figure 3C and Figure 3D). On the contrary, NF-κB expression was reduced by indirubin (Figure 3D), indicating other intracellular pathways, such as Toll-like receptors, are also involved.

### Indirubin promotes IFN-β generation through MAVS antiviral signaling after influenza infection in CORT-loaded A549 cells

**Figure 4:**
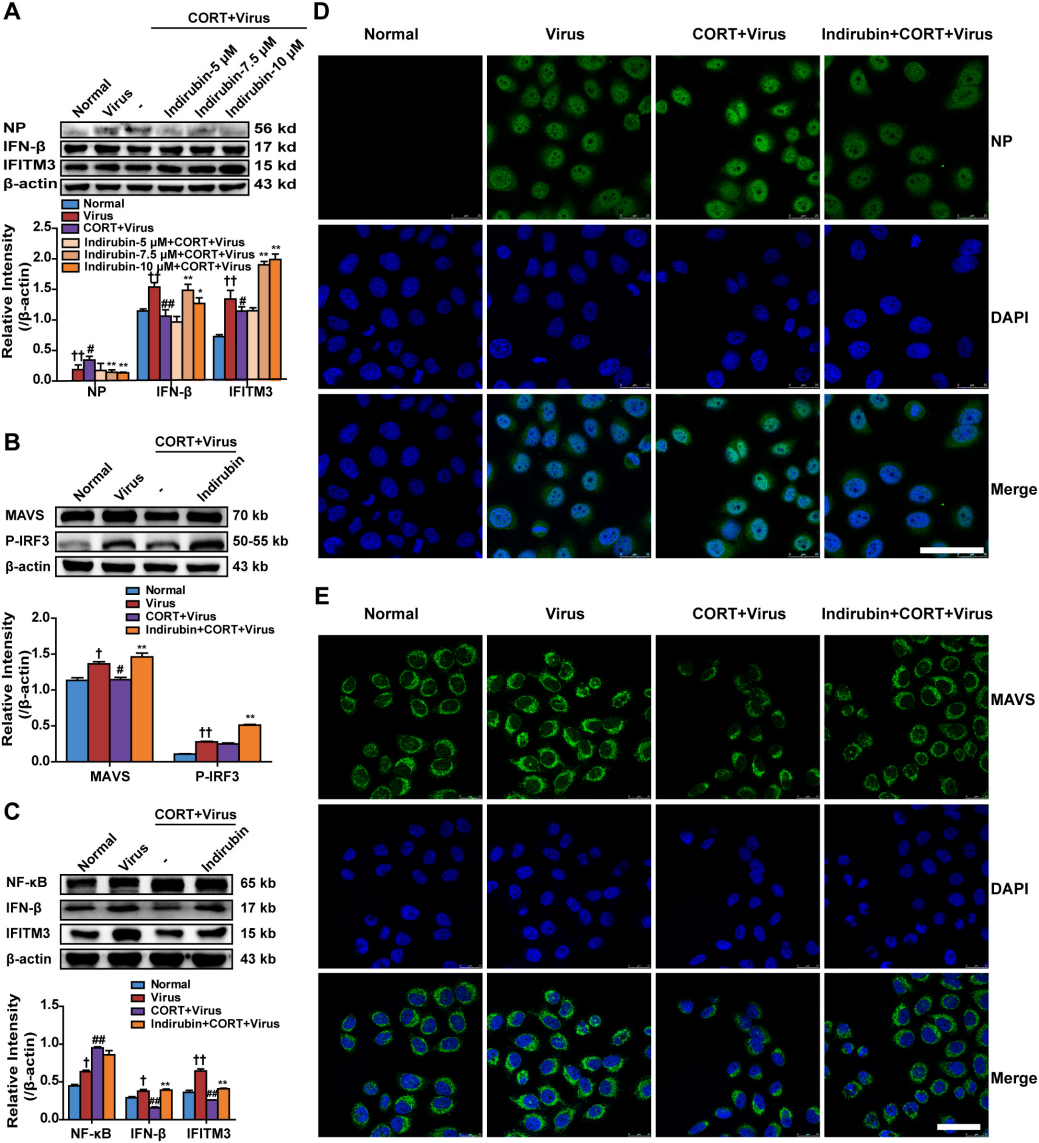
Indirubin promotes IFN-β generation through MAVS antiviral signaling after influenza infection in CORT-loaded A549 cells NP, IFN-β and IFITM3 protein expressions in CORT-loaded A549 cells treated with different concentrations of indirubin **(A)**. The protein expressions of MAVS, p-IRF3 **(B)**, NF-κB, IFN-β and IFITM3 **(C)** in A549 cells. “-” indicates no treatment. The difference was considered statistically significant at ^†^*P* < 0.05, ^††^*P* < 0.01 vs. Normal group; ^#^*P* < 0.05, ^##^*P* < 0.01 vs. Virus group; ^*^P < 0.05, ^**^P < 0.01 vs. “CORT+Virus” group. Data are expressed as mean ± SEM from 3 independent determinations. H1N1-infected CORT-loaded A549 cells were fixed and stained for NP **(D)** and MAVS **(E)** expression (representative images from n = 3 per group, NP and MAVS: green, DAPI nuclear stain: blue, scale = 50 μm).

To investigate the potential effect of indirubin on reducing influenza susceptibility *in vitro*, CORT (100 μM, 48 h) treatment was used to simulate stress in A549 cells and NP, IFN-β and IFITM3 protein levels were measured. The concentrations of 5 μM, 7.5 μM and 10 μM of indirubin were selected based on its influence on cell viability in normal cells and CORT-loaded cells (data not shown). Western blotting results showed that indirubin at 7.5 μM and 10 μM inhibited influenza virus replication and enhanced the expression of antiviral protein IFN-β and IFITM3 (Figure 4A). Then, 10 μM of indirubin was chosen to explore the influence of indirubin on MAVS antiviral signaling. The protein expressions of MAVS, p-IRF3, IFN-β and IFITM3 were remarkably declined in CORT-loaded A549 cells following influenza virus infection, while indirubin treatment improved their productions (*P* < 0.01) (Figure 4B and Figure 4C). Nevertheless, indirubin treatment reduced NP protein level in cells treated with CORT plus virus (Figure 4A). We also detected the changes of NP and MAVS levels by immunofluorescence and the results coincided with those from western blotting (Figure 4D and Figure 4E).

### Stimulator of interferon genes (STING) is involved in the regulation of IFN-β production by indirubin

**Figure 5:**
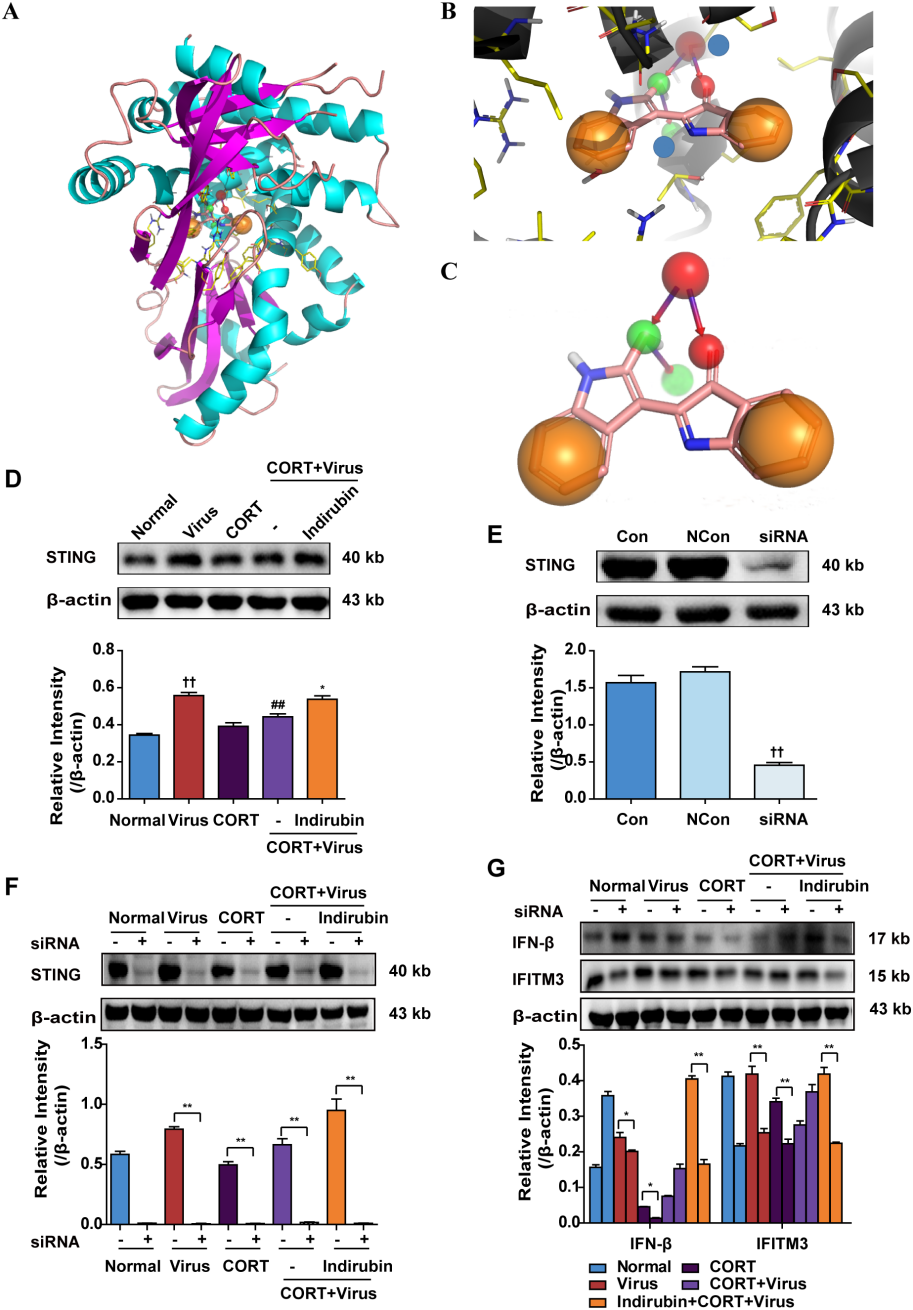
STING is involved in the regulation of IFN-β production by indirubin The structure of indirubin bound to STING **(A)**. An expanded view of the indirubin-binding pocket in the STING **(B)**. Blue spheres represented THR266 residue and THR266 residue of STING protein, respectively. Binding mode between indirubin and STING **(C)**. The protein expressions of STING **(D, E)** in A549 cells. The difference was considered statistically significant at ^††^*P* < 0.01 vs. Normal group; ^##^*P* < 0.01 vs. Virus group; ^*^P < 0.05 vs. “CORT+Virus” group. Data are expressed as mean ± SEM from 3 independent determinations. The protein expressions of STING **(F)**, IFN-β and IFITM **(G)** in A549 cells with or without siRNA transfection of STING. ^*^P < 0.05, ^**^P < 0.01. “-” indicates no treatment. Data are expressed as mean ± SEM from 3 independent determinations.

STING, a protein located in endoplasmic reticulum reticular, is well defined in the control of DNA viruses [23, 24]. Recently, its association with MAVS signaling pathway during RNA virus infection has been discovered [25, 26]. In our study, STING was predicted to be a candidate target of indirubin by Surflex-Dock program. The docking score was 6.1241, with a polar score of 1.3824 and shape similarity Tanimoto Coefficient of 0.746. As shown in Figure 5C, the carbonyls at C-3 and C-2′ of indirubin act as hydrogen bond acceptors and form an H-bond with the -NH of THR266 residue of STING. The carbonyl at C-2′ of indirubin can transform into the hydroxyl group, acting as a hydrogen bond donor, and formed an H-bond with the -N of THR262 residue of STING. In addition, the two hydrophobic groups of the benzene ring are favorable.

We then confirmed the role of STING in the anti-virus effect of indirubin using CORT-loaded A549 cell model. Results of western blotting showed that STING protein expression was increased in cells following H1N1 infection. However, it declined when cells were pre-treated with CORT, which was rescued by indirubin (10 μM) treatment (Figure 5D). On the other hand, we found that STING knockdown by the small interfering RNA (siRNA) abrogated the improving effect of indirubin on IFN-β and IFITM3 protein expression (*P* < 0.01) (Figure 5E-5G).

### The maintennance of mitochondrial morphology contributes to the effect of indirubin on MAVS signaling

**Figure 6:**
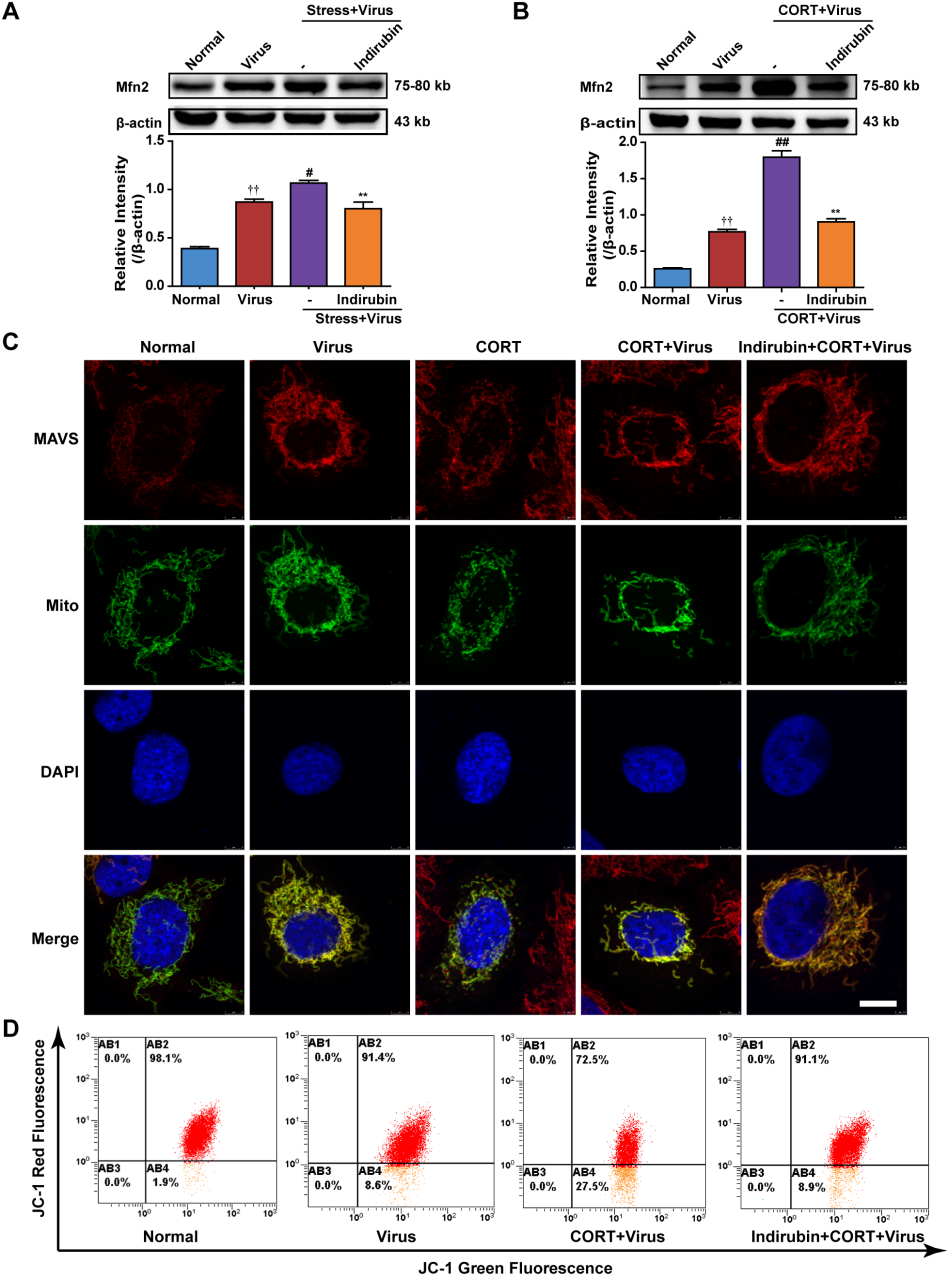
The maintenance of mitochondrial morphology contributes to the effect of indirubin on MAVS signaling Expression of Mfn2 in restraint-stressed mice **(A)** and CORT-loaded A549 cells **(B)**. The difference was considered statistically significant at ^††^*P* < 0.01 vs. Normal group; ^#^*P* < 0.05, ^##^*P* < 0.01 vs. Virus group; ^**^P < 0.01 vs. “Stress+Virus” or “CORT+Virus” group. “-” indicates no treatment. Data are expressed as mean ± SEM from 3 independent determinations. The morphology of mitochondria and the location of MAVS in A549 cells were observed by the transfection of pAcGFP1-Mito Vector and the immunofluorescence of MAVS **(C)** (MAVS: red, Mitochondria: green, DAPI nuclear stain: blue, scale = 5 μm). Mitochondrial membrane potential in A549 cells was determined by flow cytometry using JC-1 staining **(D)**.

MAVS locates the outer membrane of mitochondria and forms aggregates when it is activated. The morphology and function of mitochondria play an important role in MAVS signal transduction [22]. Mitofusin-2 (Mfn2) involves the change of mitochondrial morphology and overexpression of Mfn2 has been reported to induce small fragmented mitochondria [27]. In fact, Mfn2 has been known to counteract MAVS signaling [28]. Our data suggested that Mfn2 protein expression was increased in both the lung tissues of mice and A549 cells infected with H1N1, Intriguingly, the Mfn2 level was further promoted by stress treatment in mice or CORT treatment in A549 cells, which was prevented by indirubin administration (Figure 6A and Figure 6B). We next examined the changes of mitochondrial morphology by immunofluorescence. Confocal images indicated that small fragmented mitochondria occurred in CORT-loaded A549 cells with or without influenza virus infection, which disrupted the location of MAVS. Furthermore, indirubin treatment maintained the mitochondrial morphology and MAVS expression and location. Furthermore, the mitochondrial membrane potential (MMP) was measured by flow cytometry with JC-1 staining. As shown in Figure 6C, a remarkable increase of green fluorescence intensity in A549 cells following virus infection was aggravated by CORT pretreatment. However, indirubin (10 μM) treatment significantly prevented the decrease of MMP.

## DISCUSSION

In clinic, elder adults are susceptible to influenza virus and 90% of deaths occur in the elder population [29]. A recent study found that IAV-infected monocytes from elder humans were impaired in the production of antiviral interferons [29]. It was reported that restraint stress suppressed NK activity, decreased the number of lymphocyte cells, altered antibodies production and inflammatory response [30–32]. Our previous studies had shown restraint stress disrupted type I IFN secretion and increased the susceptibility to H1N1 influenza virus with an increased risk of dying and duration of complications [9], which paralleled to clinical features of the susceptible population [10]. The activation of hypothalamic–pituitary–adrenal axis is an important indicator of the stress response, resulting in CORT secretion [33, 34]. Studies found that CORT-induced immune suppression was the underlying mechanism of increased mortality [35]. Our previous studies have utilized CORT to establish influenza susceptibility model in A549 cells [36]. Therefore, we employed restraint-stressed mice model and CORT-loaded A549 cell model to evaluate the protective effect of indirubin on influenza susceptibility. Firstly, we evaluated its influence on the morbidity of mice caused by influenza infection in stressed mice. Results indicated that mice suffered from sickness in a certain proportion after intranasal administration of influenza virus, and restraint stress significantly aggravated the morbidity. In contrast, indirubin remarkably improved the sickness symptoms. Moreover, we also appraised the effect of indirubin on the health status of mice by their changes of body weights. Several previous reports demonstrated that after influenza virus infection, the body weights of mice decreased first and increased afterwards, although the time period of decrease depends on the type and amount of influenza virus and as well as host factors [21, 37, 38]. Our data indicated that indirubin maintained the body weights of mice in a relatively stable status, preventing a sharp drop in the early days caused by stress plus virus. Secondly, we evaluated the influence of indirubin on the mortality of mice. Results showed that indirubin significantly suppressed the decline of survival rate caused by influenza infection in stressed mice. Thirdly, the anti-influenza effect of indirubin was also noticed in the CORT-loaded A549 cell model, reflected by the reduced NP level. The above *in vitro* and *in vivo* observations demonstrated an improving effect of indirubin on reducing H1N1 susceptibility caused by stress.

Evidence has shown excessive inflammatory infiltrates and lung tissue injury are main factors accounting for high mortality following influenza virus infection [39]. Our results indicated that indirubin treatment could mitigate lung edema and inflammatory cells infiltration caused by influenza virus infection in stressed mice. When the host is infected with influenza virus, macrophages, neutrophils are recruited into the lung and produce proinflammatory cytokines to aid the immune response and limit influenza virus spread [40]. However, the cytokine storm with excessive chemokines and cytokines may lead to lung damage. Studies in animal models have found the excessive level of TNF-α contributed to the development of pulmonary lesions correlated with an acute inflammatory response [41]. IL-1β mediates acute pulmonary inflammatory pathology [42]. A report from Kaiser's lab found IL-6 was related to symptom scores and temperature values in experimental human infection [43]. IL-10, as an anti-inflammatory cytokine, mediates lung inflammation controlled by effector T cells [44]. Moreover, blocking IL-10 activity in the course of infection leads to the overproduction of proinflammatory mediators [45–47]. Our results showed that indirubin treatment lessened the production of TNF-α, IL-1β and IL-6 while enhanced the production of IL-10 in the lung tissues of influenza-infected mice loaded with stress. Our results found that indirubin exerted an anti-inflammation effect. All the results above indicated that indirubin ameliorated viral pneumonia in restraint-stressed mice.

Upon influenza virus infection, PAMPs recognize virus by PRRs and results in type I IFNs secretion [6]. Type I IFNs stimulate the expression of genes which are termed ISGs and play an important role in the antiviral state [6]. Among ISGs, IFITM3 restricts influenza A viral replication through blocking virus–host cell membrane fusion in the early step [48, 49]. In the *in vivo* studies, IFITM3 is found to be critical for protecting the host from influenza A virus, resulting in restricting the morbidity and mortality [21]. In the present study, indirubin resisted the decrease of IFN-β and IFITM3 protein expressions induced by influenza infection in stressed mice and CORT-loaded A549 cells. Then, we explored if the effect of indirubin on IFN-β production was related to the well-known anti-RNA virus pathway, namely RIG-I/MAVS/IRF3. Once detects viral RNA, RIG-I is activated and binds to MAVS located on the outer membrane of mitochondria. This interaction induces the aggregation of MAVS, followed by the activation of NF-κB and IRF3. This signaling eventually leads to the productions of pro-inflammatory cytokines and type I IFN [50]. In our study, indirubin treatment significantly restored the expression of MAVS and promoted the phosphorylation of IRF3 in stressed mice and CORT-loaded A549 cells infected with influenza virus. Unexpectedly, NF-*κ*B level was reduced by indirubin treatment, pointing out that other pathways may be involved the depressing effect on NF-*κ*B, such as Toll-like receptors or inflammasomes. Although this speculation remains to be proved, it coincides with the efficacy of indirubin on lung damage.

Upon defining the role of MAVS-mediated IFN-β generation in the anti-virus effect of indirubin, we took deeper insights into its mechanism. For such purpose, we employed Surflex-Dock program to do molecular docking and STING was found a candidate target of indirubin [51, 52]. STING resides in the endoplasmic reticulum and acts as a mitochondrial cofactor for MAVS signaling [25]. In CORT-loaded A549 cells, we utilized siRNA and western blotting techniques and confirmed the key role of STING in the activation of MAVS signaling induced by indirubin.

As the morphology and function of mitochondria are essential for the aggregation of MAVS and the interaction between MAVS and STING [25, 26], we examined the changes of mitochondria morphology and function, as well as mitochondria dynamic-related GTPase Mfn2. Confocal images and flow cytometry suggested that indirubin treatment inhibited the fragmentation of mitochondria and decline of MMP caused by influenza infection in CORT-loaded A549 cells. This finding was in line with previous two reports. The research from Koshiba's laboratory proved that MMP correlated with MAVS-mediated antiviral signaling and the dissipation in MMP reduced antiviral response [53]. Mitochondria change morphology through membrane fission and fusion, and Mfn2 plays a decisive role in the process. A previous research found mitochondrial morphology was drastically changed by overexpression of Mfn2, resulting in small fragmented mitochondria [27]. In the present study, restraint stress and CORT, respectively, induced an elevation of Mfn2 protein expression in mice and A549 cells. This change was suppressed by indirubin treatment. The results above can well explain why indirubin recovered the mitochondrial network and promoted MAVS signaling.

**Figure 7:**
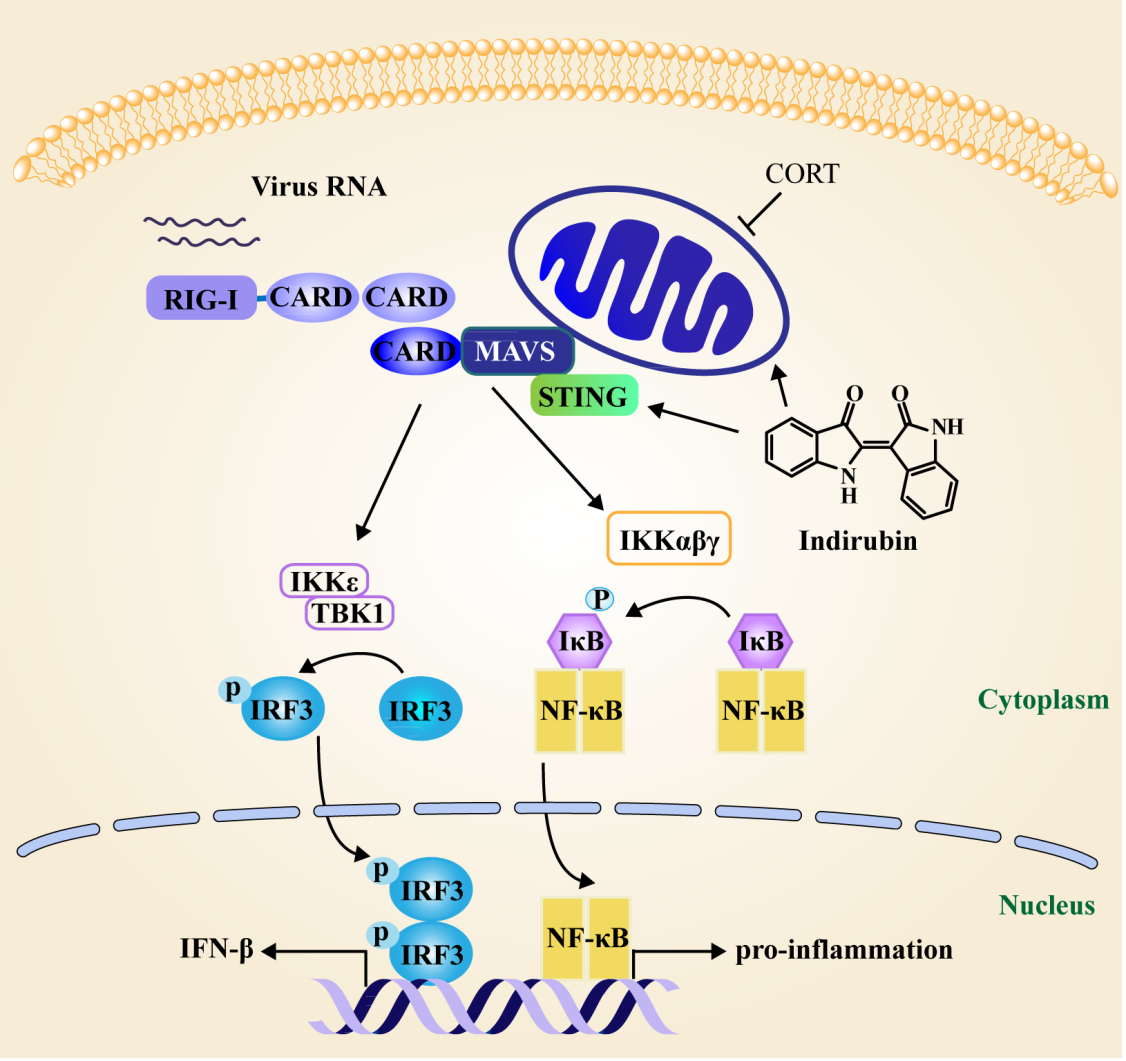
Schematic diagram of the mechanism of indirubin-induced attenuation of H1N1 pathogenesis in the susceptible model MAVS localizes to mitochondria to exert an anti-viral effect. H1N1 infection triggers host cell activation of the NF-κB and IRF3 signaling pathway following MAVS sensing. STING acts as a mitochondria cofactor for MAVS signaling to promote NF-κB and IRF3 production. CORT impacts the function of mitochondria, which has adverse effects on MAVS signaling. Indirubin promotes the production of IFN-β and IFITM3 by recovering MAVS signaling and maintaining mitochondrial function affected by CORT in the susceptible model. Moreover, indirubin is found to promote MAVS signaling by regulating STING following influenza A virus infection. In addition, indirubin attenuates NF-κB dependent pro-inflammatory cytokine production to alleviate pneumonia in restraint-stressed mice.

Overall, results have demonstrated that indirubin decreased the susceptibility to H1N1 virus and reduced the production of pro-inflammatory cytokines to alleviate pneumonia in restraint-stressed mice. Based on both restraint-stressed mice model and CORT-loaded A549 cell model, indirubin was found to maintain the morphology and function of mitochondria to ensure IFN-β production mediated by MAVS signaling following H1N1 infection Figure 7.

## MATERIALS AND METHODS

### Drugs

Indirubin (Batch No. 110717-200204) was obtained from National Institutes for Food and Drug Control (Beijing, China). Oseltamivir (Batch No. 0221301004) was purchased from Yichang Changjiang Pharmaceutical Co., Ltd. (Wuhan, China). CORT was purchased from Sigma (MO, USA).

### Virus

Influenza A/FM/1/47 (H1N1) virus was donated by Prof. Jianxin Chen in South China Agricultural University (Guangzhou, China). New viral stocks were passaged in the allantoic cavities of specific pathogen-free fertilized eggs. Then, virus fluid was kept at −80°C before use. The median lethal dose (LD_50_) was measured in mice and a 2×LD_50_ amount was applied for the animal experiments related to influenza virus. The median tissue culture infective dose (TCID_50_) was measured in A549 cells and 10 TCID_50_ amount was applied to the *in vitro* experiments.

### Mice and treatments

Specific-pathogen-free male Kunming mice (three-week-old, 13-15 g) were obtained from Guangdong Medical Laboratory Animal Center (Guangzhou, China, Approval ID: SCXK 2013-0002). The animals were raised in plastic cages with bedding material of corn straw and lived at 23 ± 2°C with a cycle of equal light and dark. The relative humidity was 50 ± 10%. Animal experiments conformed to the Animal Care and Use Committee of Jinan University (Approval No: 20150310001), as well as the National Institute of Health's Guide for the Care and Use of Laboratory Animals (7^th^ edition, USA).

In the first animal experiment, mice were distributed at random to six groups: Normal, Virus, “Stress+Virus”, “Oseltamivir+Stress+Virus” (30 mg·kg^−1^·d^−1^ oseltamivir), and two indirubin (5 or 2.5 mg·kg^−1^·d^−1^) groups which are described as “Indirubin-H+Stress+Virus” and “Indirubin-L+Stress+Virus” respectively. Oseltamivir and indirubin were administered orally to mice for seven days, while mice in groups were orally treated with equal volume of water. On the first day of administration, mice except those in Normal and Virus groups were restricted in ventilated plastic centrifuge tubes of 50 mL for 22 h. On the 3^rd^ day, mice were anesthetized using diethyl ether vapor and immediately infected with influenza A virus suspension (2×LD_50_) in PBS of 35 μL. Subsequently, the daily changes of mice in survival, body weight, typical influenza symptoms, such as hunched back, ruffled fur, altered respiration and unresponsiveness, were observed and recorded for 21 days or until death. Consequently, the data of morbidity rate and survival rate were calculated.

The second animal experiment was also conducted in restraint-stressed mice. The effect of indirubin on pneumonia caused by influenza virus was evaluated. The animal grouping is the same as the first experiment. However, five days post virus infection, the weights of mice were recorded. Subsequently, mice were anesthetized using ether vapor and sacrificed to harvest lungs for histopathologic examination and western blotting analysis of inflammation mediators. The lungs were weighted and lung index was counted in accordance with the formula: Lung index (mg·g^−1^) = lung weight/body weight.

The third animal experiment was conducted to investigate the effect and mechanism of indirubin on type I IFN secretion in stressed mice. Mice were distributed at random to four groups: Normal, Virus, “Stress+Virus” and “Indirubin+Stress+Virus” (5 mg·kg^−1^ indirubin). The following treatment was the same as the second experiment. The lung tissues were collected to determine the protein expressions related with IFN-β and MAVS signaling.

### Cell culture and treatment

A549 cells, a carcinomic human alveolar basal epithelial cell line, were cultured in Dulbecco's Modified Eagle's Medium (DMEM) (Biological Industries, CT, USA) containing 10% heat-inactivated fetal bovine serum (FBS) (Biological Industries, CT, USA). The CO_2_ concentration of incubator was 5% and the temperature was at 37°C.

Cells were randomly divided into four groups: Normal, Virus, “CORT+Virus”, “Indirubin+CORT+Virus”. Cells were seeded onto cell culture dish in 8×10^4^ cells·ml^−1^ for experiments. After one day, the cells were cultured with CORT (100 μM) or CORT (100 μM) plus indirubin (10 μM) for 48 h and were subsequently overlaid with H1N1 influenza virus of 10 TCID_50_ for 45 min at 4°C, then PBS was used to clean cells for two times and serum-free DMEM was added. Eight hours post influenza virus infection, the cells were collected for the determination of protein expression.

### Lung histopathology

Lungs were removed and fixed in 4% paraformaldehyde with a hand-held 5 mL syringe to pump out air. Then the lung tissue was embedded in paraffin wax and sliced in 4 μm thickness. The slices were stained with H&E. Histopathologic changes were observed using ×10 objectives (Olympus, DP70, Japan).

### Western blotting analysis

For western blotting analysis, the proteins of lung samples and cell lysates lysed by RIPA buffer (Beyotime, Shanghai, China) were separated using SDS-polyacrylamide gel electrophoresis. Subsequently, the proteins resolved in the gel were transferred to the polyvinylidene fluoride membrane (Millipore, MA, USA). Western blots were visualized using the ECL system (Fdbio Science, Hangzhou, China) and Quantity One software (Bio-Rad, USA) was used to analyze the intensity of individual bands. Data were normalized with β-actin as a loading control in each individual sample. The following antibodies were used in western blotting analysis: influenza virus NP rabbit pAb (1:2000), IFITM3 rabbit pAb (1:2000), IL-1β rabbit pAb (1:1200) and IL-6 rabbit pAb (1:1200) were provided by Abcam (MA, USA). Mfn2 mouse mAb (1:2,000) and IL-10 mouse mAb (1:2000) were obtained from Santa Cruz Biotechnology (CA, USA). β-actin rabbit pAb (1:2000) was from Bioworld (MN, USA). IFN-β rabbit pAb (1:500) was from OriGene Technology (MD, USA). p-IRF3 rabbit mAb (1:1000), NF-κB p65 rabbit mAb (1:2000) and TNF-α rabbit pAb (1:2000) were provided by Cell Signaling Technology (MA, USA). MAVS rabbit pAb (1:500) and STING rabbit pAb (1:500) antibodies were obtained from Proteintech Group (IL, USA).

### Cell immunofluorescence

Cells were fixed in 4% paraformaldehyde for 15 min, permeabilized with 0.1% Triton-X100 for 5 min, then either stained with a rabbit polyclonal antibody against NP (Abcam, MA, USA) or a rabbit polyclonal antibody against MAVS (Proteintech Group, IL, USA) in 1:50 dilution at 4°C for 12 h. Cells were then incubated with Alexa Fluor 488 conjugated goat anti-rabbit IgG (Life Technology, NY, USA) in 1:500 dilution. 4′, 6-diamidino-2-phenylindole (DAPI, 5 μg·ml^−1^, Beyotime, Shanghai, China) was used to stain cell nuclei. Cells were observed and analyzed using a confocal laser scanning microscope (Leica, TCS SP8, Germany).

### Molecular docking

The Surflex-Dock program implemented in SYBYL-8.1 (http://bioinfo-pharma.u-strasbg.fr/scPDB/) was used to dock the indirubin into protein structures from the scPDB database (http://cheminfo.u-strasbg.fr/scPDB). This database contains 9283 protein structures, including 3678 unique targets [45]. Docking was performed using the default parameter in pgeom mode. Gaussian shape similarity between each docking pose and the cognate ligand were also evaluated using shape-it program (Version 1.0.1,http://silicos-it.be.s3-website-eu-west-1.amazonaws.com). Docking poses with a polar score of smaller than 0.8 and shape similarity Tanimoto coefficient of smaller than 0.7 were discarded.

### SiRNA transfection

The siRNA transfection was performed using targeting sequences 5′-GAAGAGGUAUUGAAUGCUA-3′ and 5′-CUUCUCCAUAACUUACGAU-3′ against STING (Ribo Bio, Guangzhou, China). A non-targeting siRNA (Ribo Bio, Guangzhou, China) was used as negative control. The transfection, using lipofectamine 2000 (Invitrogen, NY, USA), was carried out as described in the instructions of the manufacturer. Six hours after transfection, cells were incubated with vehicle, CORT (100 μM), or indirubin (10 μM, added 2 h before CORT) plus CORT (100 μM) for 48 h. Cells were then overlaid with 10 TCID_50_ H1N1 virus as before. Eight hours later, the cells were harvested and lysed for the determination of protein expressions.

### pAcGFP1-Mito transfection

A549 cells were cultured on glass bottom cell culture dish of 15 mm (NEST Biotech, Wuxi, China) in 8×10^4^ cells·ml^−1^ and subsequently prepared for pAcGFP1-Mito (Clontech, CA, USA) transfection to reach 50-60% confluence. A non-targeting vector (Clontech, CA, USA) was used as negative control. The transfection, using lipofectamine 2000 (Invitrogen, UY, USA), was carried out as described in the instructions of the manufacturer. Six hours after transfection, cells were incubated with CORT or CORT plus indirubin for 48 h. Subsequently, cells were overlaid with 10 TCID_50_ H1N1 virus as before. Eight hours later, cells were stained with MAVS rabbit pAb in 1:50 dilution at 4°C for 12 h and then stained with Alexa Fluor 555 conjugated goat anti-rabbit IgG (Life Technology, NY, USA) in 1:500 dilution. Cell nuclei were stained using DAPI (5 μg·ml^−1^) (Beyotime, Shanghai, China). The fluorescence was analyzed using a confocal laser scanning microscope (Leica, TCS SP8, Germany).

### MMP detection

JC-1 probe (Sigma, MO, USA) was used to detect MMP of A549 cells. After harvested, cells were incubated with the JC-1 solution (10 μg·ml^−1^) at 37°C for 20 min. Subsequently, cells were cleaned with PBS, resuspended in 1 mL of sheath fluid and filtered through a 40 μm nylon mesh. Cells were then harvested and analyzed by detecting the JC-1 fluorescence emission using a flow cytometry (Beckman, Epics XL, USA).

### Statistical analysis

These studies complied with the recommendations on experimental design and analysis in pharmacology [50]. All data were expressed as means ± SEM. At least five different experiments were conducted to obtain the data. The statistical differences between the groups were estimated (SPSS, Version 15, USA) using ANOVA for multiple comparisons with Tukey post-hoc test to determine statistical significance. Survival curves were assessed using Kaplan-Meier method and the group differences were estimated using the log-rank test of GraphPad Prism5 (GraphPad Software, CA, USA). Differences were considered significant when the P value was less than 0.05. All statistical tests were two-sided.

## Abbreviation

CORT: corticosterone
DAPI: 4′, 6-diamidino-2-phenylindole
DMEM: Dulbecco's Modified Eagle's Medium
FBS: fetal bovine serum
H&E: hematoxylin and eosin
IFITM3: interferon inducible transmembrane 3
IFN: interferon
IL: interleukin
ISGs: IFN-stimulated genes
LD50: median lethal dose
MAVS: mitochondrial antiviral signaling
Mfn2: mitofusin-2
MMP: mitochondrial membrane potential
NA: neuraminidase
NP: nucleoprotein
PAMPs: pathogen-associated molecular patterns
p-IRF3: phosphorylation interferon regulatory factor 3
PRRs: pattern recognition receptors
RIG-I: retinoic acid-inducible gene I
siRNA: small interfering RNA
STING: stimulator of interferon genes
TCID50: median tissue culture infective dose
